# Suppressing the Na^+^/H^+^ exchanger 1: a new sight to treat depression

**DOI:** 10.1038/s41419-019-1602-5

**Published:** 2019-05-08

**Authors:** Xueyang Deng, Zhouye Ji, Bingru Xu, Liting Guo, Lixing Xu, Tingting Qin, Liang Feng, Zhanqiang Ma, Qiang Fu, Rong Qu, Qinglong Quo, Shiping Ma

**Affiliations:** 1 Department of Pharmacology of Chinese Materia Medica, China Pharmaceutical University, 210009 Nanjing, China; 2 Qinba Traditional Chinese Medicine Resources Research and Development Center, AnKang University, 725000 AnKang, PR China; 3 Department of Pharmacology of Traditional Chinese Medical Formulae, Nanjing University of Traditional Chinese Medicine, 210029 Nanjing, China; 4 State Key Laboratory of Natural Medicines, Jiangsu Key Laboratory of Carcinogenesis and Intervention, China Pharmaceutical University, 210009 Nanjing, China; 5 School of Traditional Chinese Pharmacy, China Pharmaceutical University, 211198 Nanjing, Jiangsu China

**Keywords:** Pharmacology, Cellular neuroscience, Dementia

## Abstract

Na^+^/H^+^ exchanger 1 (NHE1), an important regulator of intracellular pH (pHi) and extracellular pH (pHe), plays a crucial role in various physiological and pathological processes. However, the role of NHE1 in depression has not yet been reported. This study was designed to investigate the role of NHE1 in the animal model of depression and explore the underlying mechanisms. Our results showed that inhibition of rho-associated kinase 2 (ROCK2) by fasudil (Fas) or baicalin (BA) significantly alleviated chronic unpredictable mild stress (CUMS) paradigm-induced depression-related behaviours in mice, as shown by decreased sucrose consumption in sucrose preference test (SPT), reduced locomotor activity in the open field test (OFT), and increased immobility time in the tail suspension test (TST) and forced swimming test (FST). Furthermore, ROCK2 inhibition inhibited the activation of NHE1, calpain1, and reduced neuronal apoptosis in the CUMS animal model of depression. Next, we used the lipopolysaccharide (LPS)-challenged animal model of depression to induce NHE1 activation. Our results revealed that mice subjected to 1 μl LPS (10 mg/ml) injection intracerebroventricularly (i.c.v.) showed depressive-like behaviours and NHE1 activation. Amiloride (Ami), an NHE1 inhibitor, significantly reversed the decrease in sucrose consumption and reduction in immobility time in the TST and FST induced by LPS challenge. Furthermore, Ami decreased the expression of ROCK2, NHE1, calpain1, and caspase-3 and increased the Bcl-1/Bax ratio in the hippocampus of LPS-challenged mice. Ami treatment also led to antidepressive effects in the CUMS-induced animal model of depression. Thus ROCK2 inhibition could be proposed as a neuroprotective strategy against neuronal apoptosis, and NHE1 might be a potential therapeutic target in depression.

## Introduction

Neurons in the central nervous system (CNS) regulate their intracellular pH (pHi) via particular membrane proteins. Na^+^/H^+^ exchanger (NHE) is a primary membrane protein ubiquitously expressed with which neurons adjust their pHi to mediate DNA synthesis, cell volume, and protein function and degradation in the initiation of cellular growth and differentiation^1,2^. As the most widely expressed isoform in NHE family, NHE1 plays an important role in regulating the physiological and pathophysiological process in diseases of the central nervous system^3–5^; however, the role of NHE1 in depression has not yet been reported. Associations between the function of NHE1 and the pathogenesis of depression, such as impaired neurotransmitter release, elevated central inflammation, and hyperactive hypothalamic–pituitary–adrenal (HPA) axis, has been described by recent studies. For example, NHE1 regulates pHi and extracellular pH changes that contribute in part to the hydrogen ion sensitivity of voltage-gated ion channels, as well as neurotransmitter receptors^6^. The central neurotransmitter, 5-hydroxytryptamine (5-HT), enhances intestinal NHE activity via stimulation of the G-coupled 5-HT_1A_ and G_q/11_-coupled 5-HT_2_ receptors^7^. Altered NHE1 function influences neuronal excitability and plays a role in epilepsy. Major depression is also recognised as a chronic inflammatory neuropsychiatric disorder^8^.

Rho-associated kinase (ROCK) plays an important role during the treatment of various diseases, including CNS disorders. ROCK has two isoforms, ROCK1 and ROCK2. ROCK2 is preferentially expressed in the brain and muscle, whereas ROCK1 is primarily expressed in the non-neuronal organs^9^. ROCK2 is a major regulator of axonal degeneration, neuronal death, and axonal regeneration in the CNS. Research has reported that ROCK2, instead of ROCK1, is the relevant isoform in acute ischaemic stroke and ROCK2-related signalling is suggested to play a key role in the animal model of depression^10^. More importantly, ROCK2 is also reported as an important upstream regulator of NHE1. A recent study stated that NHE1 was identified as a potential target of ROCK signalling in response to lysophosphatidic acid treatment^11^. The pharmacological inhibition of ROCK blocked the increase of NHE1 function in astrocytoma cells^12^. In addition, using mass spectrometry and reconstituted kinase assays, ROCK1 and ROCK2 stoichiometrically phosphorylate NHE1 at threonine-653 in vitro^11^. Furthermore, at pathological levels, the deregulation of NHE1 is responsible for alterations in pHi, intracellular Ca^2+^ aggregation, proliferation, and apoptosis^13^. These observations suggest that ROCK2 be required for the hyperactivity of NHE1.

The association of NHE1 and inflammation has been characterised both in vitro and in vivo. NHE1 was reported to play an important role reducing inflammatory pain in the dorsal root ganglion and spinal cord in rats^14^. NHE1 inhibition reduced the inflammatory responses and lessened myocardial, liver, and kidney injuries by reducing nuclear factor-κB activation and induced nitric oxide synthase expression, as well as attenuating extracellular signal-regulated kinase 1/2 phosphorylation^15^. Meanwhile, NHE1 activation affected the import of Na^+^ that alters the intracellular milieu, triggering the activation of several other transporters. Increased [Na^+^]i stimulates the activation of several transporters, such as the Na^+^/K^+^ ATPase and Na^+^/Ca^2+^, leading to the activation of a variety of downstream molecular changes^16,17^. In this manner, NHE1 alters cellular function both by regulating the pH and stimulating the intracellular signalling cascades. Thus we proposed that NHE1 might play an important role in the pathogenesis of depression, and this study was designed to explore NHE1-associated signalling in an animal model of depression.

## Results

### Chronic unpredictable mild stress (CUMS)-induced depressive-like behaviours in mice were reversed by ROCK2 inhibition

**Fig. 1:**
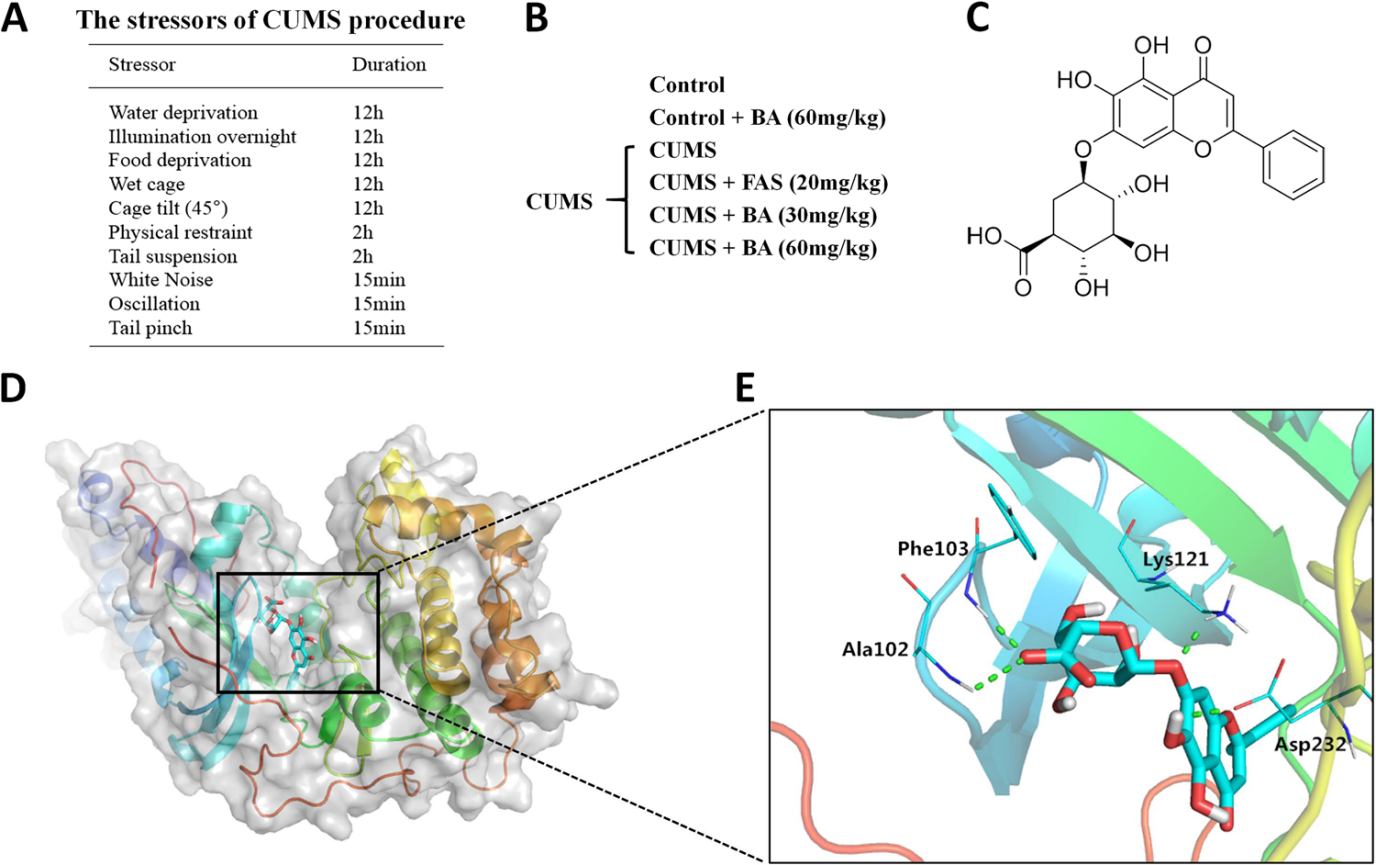
Chronic unpredictable mild stress (CUMS) paradigm and drug treatment. **a** The stressors in the CUMS paradigm. **b** The drug treatment in the CUMS procedure. **c** The structure of baicalin (BA). **d**, **e** Molecular docking analyses of the interaction between BA and Rho-associated kinase 2

**Fig. 2:**
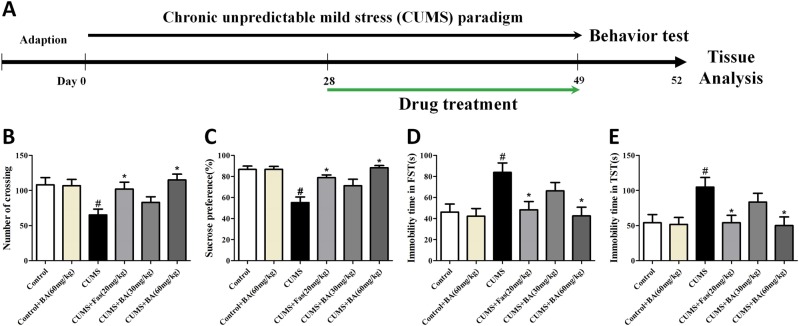
Chronic unpredictable mild stress (CUMS)-induced depressive-like behaviours in mice were reversed by chronic Rho-associated kinase 2 (ROCK2) inhibitor treatment. **a** Schematic diagram of the experimental design for the CUMS paradigm. **b** Effects of ROCK inhibition on the number of crossing in CUMS-exposed mice in the open field test. **c** Effects of ROCK2 inhibition on the sucrose preference percentage in CUMS-exposed mice. **d** Effects of ROCK2 inhibition on the immobility time in CUMS-exposed mice in the forced swimming test. **e** Effects of ROCK inhibition on the immobility time in CUMS-exposed mice in the tail suspension test. Values are expressed as means ± SEM. ^#^*p* < 0.05 vs. control group; *p < 0.05 vs. CUMS group

The effects of ROCK2 inhibition were evaluated in the CUMS-induced animal model of depression by applying fasudil (Fas, 20 mg/kg) and baicalin (BA, 30 or 60 mg/kg) (Figs. 1b and 2a). To determine how BA structurally inhibited ROCK2, we performed molecular docking of BA to ROCK2 using the Autodock program. The structure of BA is shown in Fig. 1c, and the docking results are shown in Fig. 1d–e. BA binds to ROCK2 by forming stable hydrogen bonds at Phe103, Ala102, Lys121, and Asp232. The binding energy of BA to ROCK2 was −6.09 kcal/mol.

### Effects of ROCK2 inhibition on open field test (OFT) and sucrose preference test (SPT) results in CUMS-exposed mice

The effect of ROCK2 inhibition on the spontaneous locomotor activities of mice was tested in the OFT. As shown in Fig. 2b, the number of crossings was significantly lower in the CUMS group than in the control group. Mice in the CUMS+Fas (20 mg/kg) or CUMS+BA (60 mg/kg) groups exhibited a significantly higher number of crossings than those in the CUMS group. Furthermore, there was no difference in the number of crossings between the control+BA (60 mg/kg) and control groups, indicating that BA produced an antidepressive effect without affecting central excitability. As exhibited in Fig. 2c, sucrose consumption was reduced after CUMS exposure, whereas the CUMS+BA (60 mg/kg) and CUMS+Fas (20 mg/kg) groups showed significant improvements in sucrose consumption.

### Effects of ROCK2 inhibition on immobility time in the forced swimming test (FST) and tail suspension test (TST) in CUMS-exposed mice

We then examined the effects of ROCK2 inhibition on the behaviours in the FST and TST in mice (Fig. 2d, e). Animals in the CUMS group, compared to those in the control group, exhibited increased immobility time in the FST. Nevertheless, Fas (20 mg/kg) or BA treatment (60 mg/kg) significantly attenuated the CUMS-induced increase in immobility time in the FST. The results in Fig. 2e show that mice in the CUMS group, in comparison to those in the control group, had increased immobility time in the TST. Mice in the CUMS+Fas (20 mg/kg) and CUMS+BA (60 mg/kg) groups showed decreased immobility time in the TST.

### Effects of ROCK2 inhibition on neuronal apoptosis in the hippocampus of CUMS-exposed mice

**Fig. 3:**
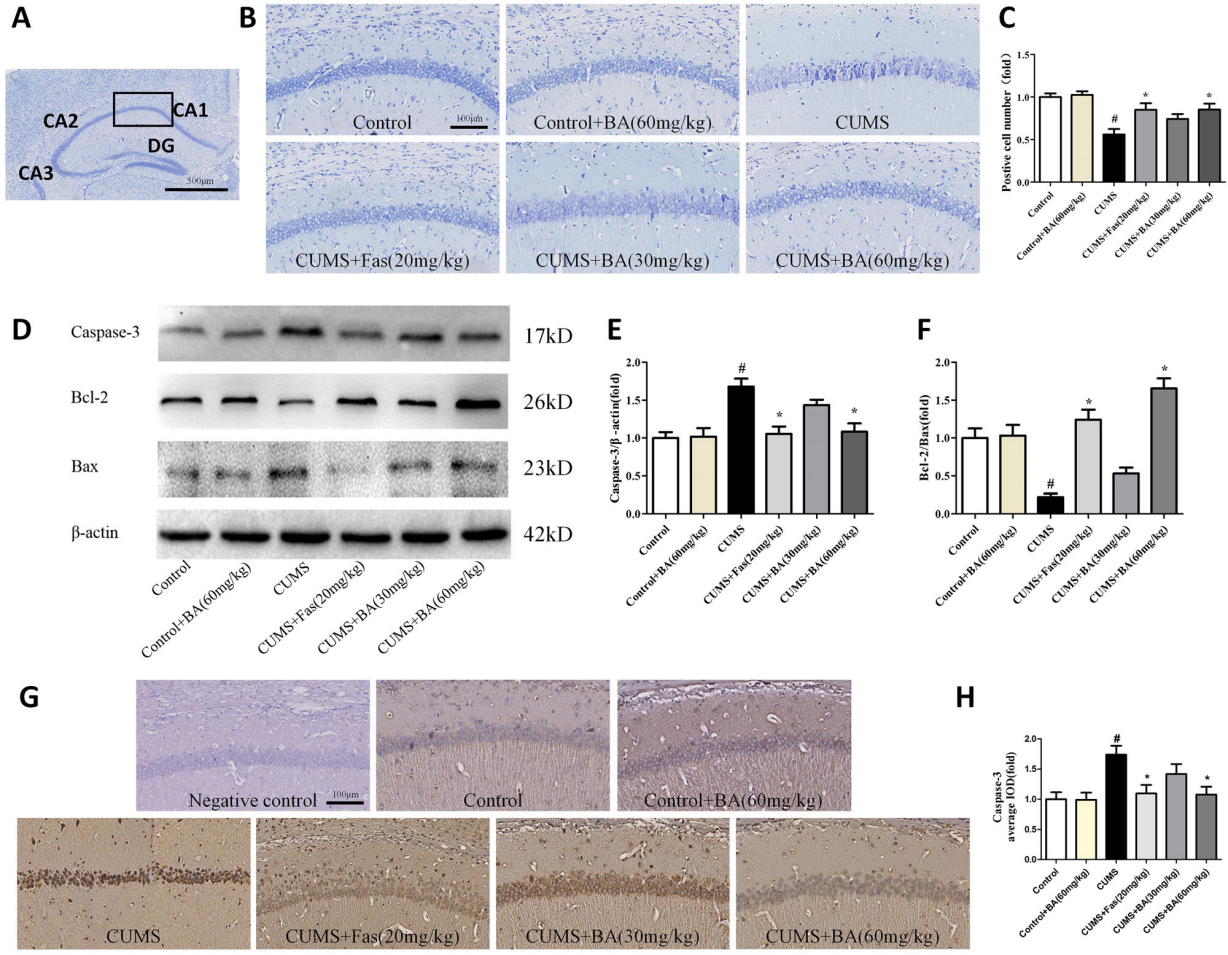
ROCK2 inhibition attenuated neuronal apoptosis in the hippocampus of chronic unpredictable mild stress (CUMS)-exposed mice. **a** The location of the CA1 region to be evaluated both in the Nissl and immunohistochemical experiments marked by a rectangle with a black border (magnification ×5, scale bar = 500 μm). **b** Representative photographs of Nissl-stained hippocampal CA1 region (magnification ×20, scale bar = 100 μm). **c** Quantitative analysis of Nissl bodies in hippocampal CA1 region. **d** Representative western blot for caspase-3, Bcl-2, and Bax expression. **e**, **f** The expression of caspase-3, and the Bcl-2 to Bax ratio. **g**, **h** The immunohistochemical staining of caspase-3 in the CA1 region of the hippocampus (scale bar = 100 μm). Values are expressed as means ± SEM. ^#^*p* < 0.05 vs. control group; *p < 0.05 vs. CUMS group

The location of the CA1 region to be evaluated in the Nissl and immunohistochemical experiments was indicated in Fig. 3a, which was marked by a rectangle with a black border. As shown in Fig. 3b, c, in the hippocampal CA1 region of CUMS-exposed mice, the number of Nissl-positive cells were significantly lower. Mice in the CUMS+BA (60 mg/kg) and CUMS+Fas (20 mg/kg) groups showed a remarkably higher number of Nissl-positive cells. Immunoblotting tests indicated that mice in the CUMS+BA (60 mg/kg) and CUMS+Fas (20 mg/kg) exhibited decreased caspase-3 and increased Bcl-2/Bax ratio relative to the CUMS group (Fig. 3d–f). These data indicate that ROCK2 inhibition prevented neural apoptosis. In addition, immunohistochemical examination further showed that CUMS exposure significantly increased the expression of caspase-3 in the hippocampal CA1 region relative to those in the control group (Fig. 3g, h). These results suggested that ROCK2 inhibition attenuated hippocampal neuronal apoptosis in the CUMS-induced animal model of depression.

### Effects of ROCK2 inhibition on NHE1-related pathways in the hippocampus of CUMS-exposed mice

**Fig. 4:**
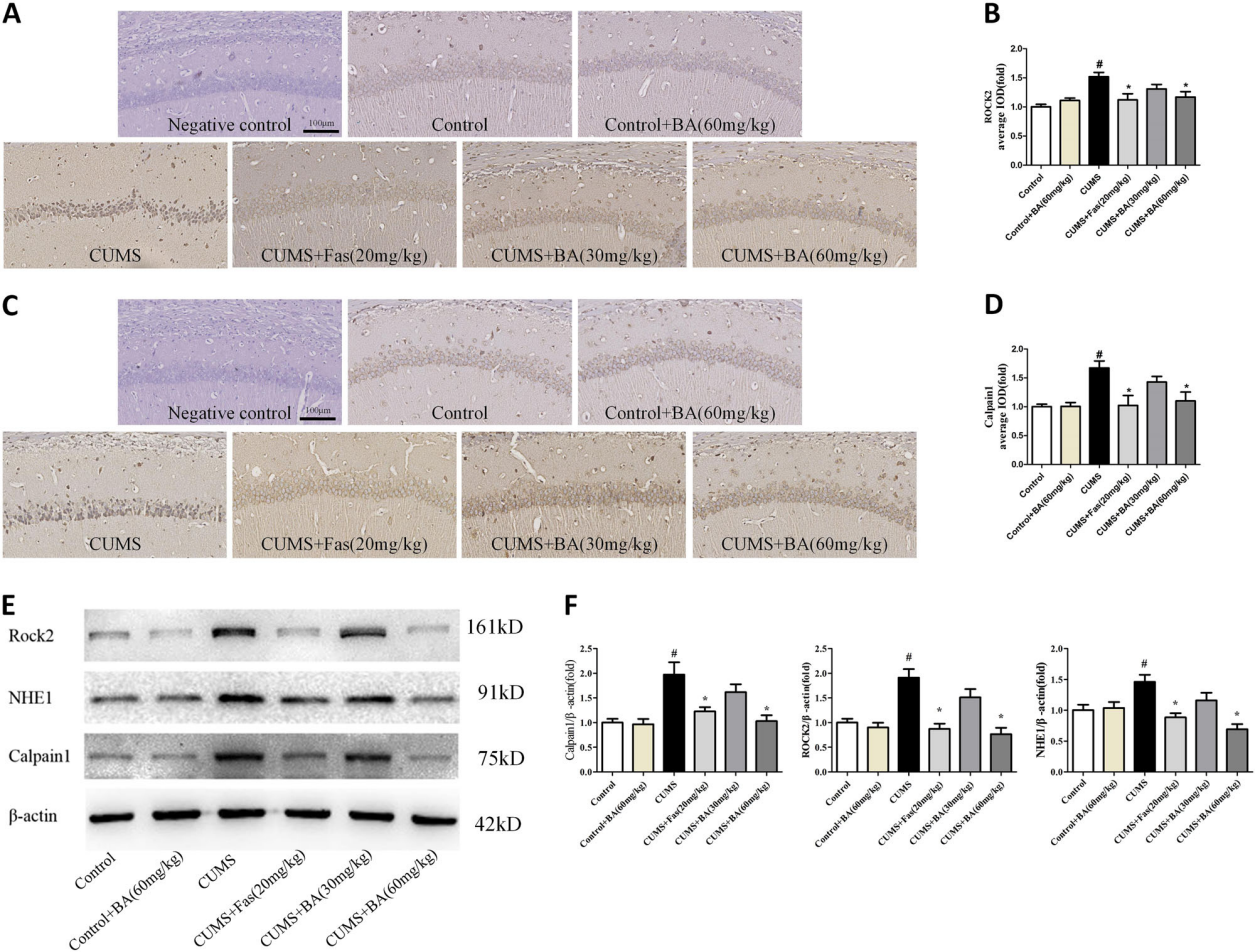
Rho-associated kinase 2 (ROCK2) inhibition attenuated the Na^+^/H^+^ exchanger 1 (NHE1)-regulated pathway in the hippocampus of chronic unpredictable mild stress (CUMS)-exposed mice. **a**, **b** Immunohistochemical staining and quantitative analysis of ROCK2 in the hippocampal CA1 region of CUMS-exposed mice. **c**, **d** Immunohistochemical staining and quantitative analysis of calpain1 in the CA1 region of the hippocampus. **e** Representative western blot of ROCK2, NHE1, and calpain1. **f** The relative expression of ROCK2, NHE1, and calpain1 in the western blot experiment. Scale bar = 100 μm. Values are expressed as means ± SEM. ^#^*p* < 0.05 vs. control group; *p < 0.05 vs. CUMS group

To elucidate the underlying mechanism of ROCK2 inhibition on repressing apoptosis, we determined the levels of NHE1-related signalling. ROCK2 expression in the CUMS-exposed mice was significantly higher than that in the control group. However, CUMS+BA (60 mg/kg) and CUMS+Fas (20 mg/kg) treatment reversed this increase relative to the CUMS group (Fig. 4a, b). Furthermore, the CUMS group exhibited a remarkable elevation in the expression of calpain1, whereas CUMS+BA (60 mg/kg) and CUMS+Fas (20 mg/kg) treatment downregulated calpain1 expression, as shown in (Fig. 4c, d). From the results shown in Fig. 4e, f, the CUMS group exhibited a notable elevation in the expression of NHE1, calpain1, and ROCK2 relative to the control group. CUMS+BA (60 mg/kg) and CUMS+Fas (20 mg/kg) treatment significantly reversed the expression of NHE1-related signalling relative to the CUMS group.

### Effects of NHE1 inhibition on the LPS-induced animal model of depression

**Fig. 5:**
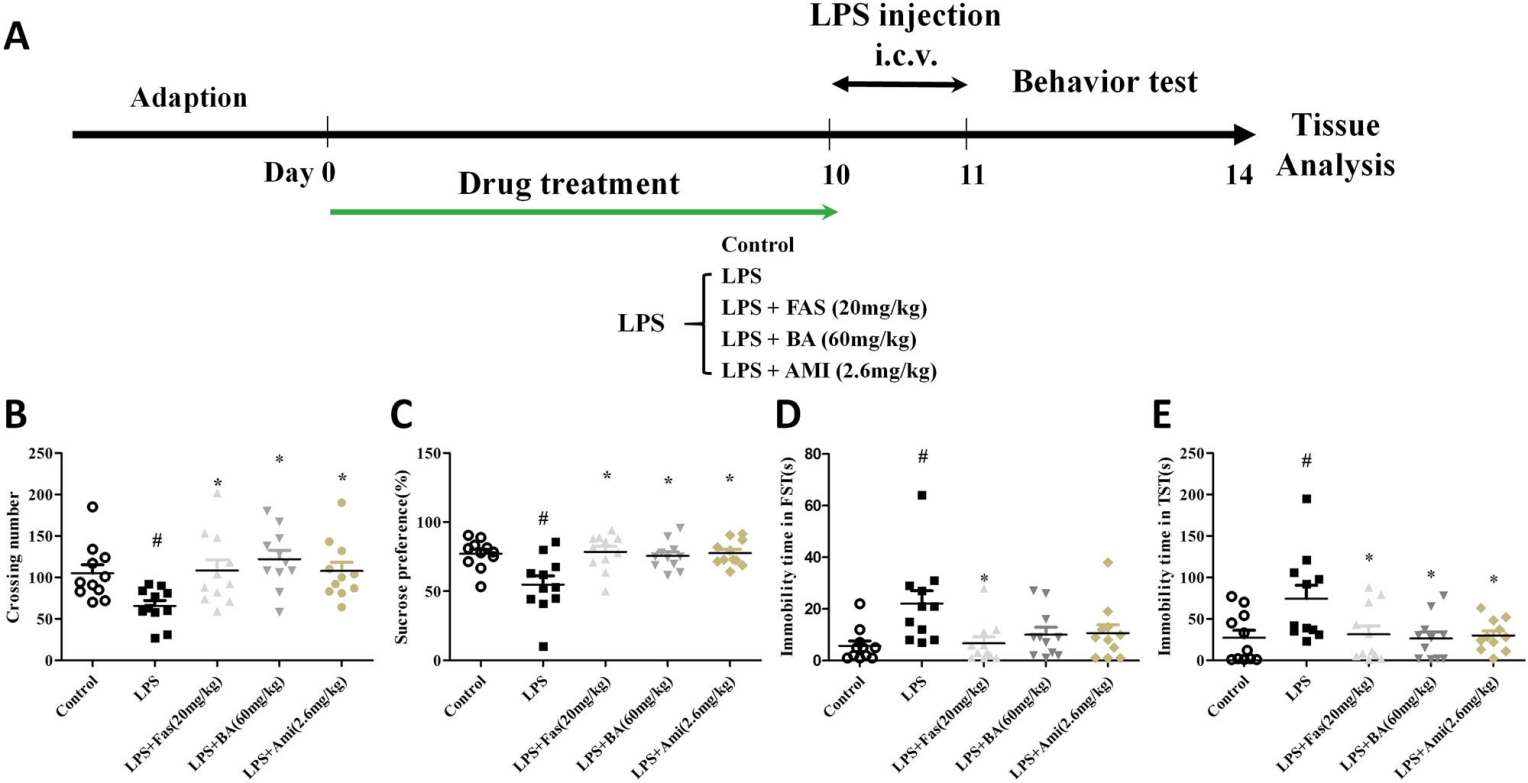
Lipopolysaccharide (LPS)-induced depressive behaviours in mice were reversed by Na^+^/H^+^ exchanger 1 (NHE1) inhibition. **a** The experimental design and drug treatment for the LPS paradigm. **b** Effects of NHE1 inhibition by amiloride (Ami; 2.6 mg/kg), baicalin (BA; 60 mg/kg), and fasudil (Fas; 20 mg/kg) on the number of crossings performed by LPS-treated mice in the open field test. **c** Effects of Ami (2.6 mg/kg), BA (60 mg/kg), and Fas (20 mg/kg) on the sucrose preference percentage of LPS-treated mice. **d** Effects of NHE1 inhibition on the immobility time in LPS-treated mice in the forced swim test. **e** Effects of NHE1 inhibition on the immobility time in LPS-treated mice in the tail suspension test. Values are expressed as means ± SEM. ^#^*p* < 0.05 vs. control group; *p < 0.05 vs. LPS group

To further demonstrate the pathogenesis of NHE1-regulated depression, we employed LPS to induce depressive-like symptoms in mice^18,19^ because LPS could activate NHE1, which is related to the activation of ROCK2 and excessive aggregation of Ca^2+^ in the cytoplasm. Furthermore, LPS treatment triggers inflammatory responses, leading to neuronal apoptosis, which is also a well-established animal model of depression. Amiloride (Ami), as the inhibitor of NHE1, was administered to LPS-exposed mice, and the experimental design is as shown in Fig. 5a.

### LPS-induced depressive behaviours in mice were reversed by NHE1 inhibition

We examined the spontaneous locomotor activity after LPS exposure. The OFT is shown in Fig. 5b; a lower number of crossings was exhibited in the LPS group than in the control group. Ami (2.6 mg/kg), BA (60 mg/kg), and Fas (20 mg/kg) treatment improved the behavioural changes relative to the LPS group (Fig. 5b). As shown in Fig. 5c, mice in the LPS group, in comparison with those in the control group, exhibited a significant decrease in sucrose consumption. Pre-treatment with Ami (2.6 mg/kg) exerted an antidepressant-like effect on LPS-exposed mice as sucrose consumption significantly increased. Pre-treatment with BA (60 mg/kg) and Fas (20 mg/kg) exhibited the same effects on sucrose consumption in mice exposed to LPS.

### Effects of NHE1 inhibition in the FST and TST in LPS-treated mice

We then tested the effects of drug administration in the FST. As shown in Fig. 5d, immobility time in the FST was significantly increased in the LPS-exposed group relative to the control group. Meanwhile, the effect was reversed by pre-treatment with Fas (20 mg/kg). Finally, we tested the effects of Ami (2.6 mg/kg), BA (60 mg/kg), and Fas (20 mg/kg) administration in the TST. As shown in Fig. 5e, immobility time in the FST was significantly increased in the LPS exposure group relative to the control group. Meanwhile, the effect was reversed by pre-treatment with Ami (2.6 mg/kg), BA (60 mg/kg), and Fas (20 mg/kg).

### Effects of NHE1 inhibition on LPS-induced inflammation and apoptosis

**Fig. 6:**
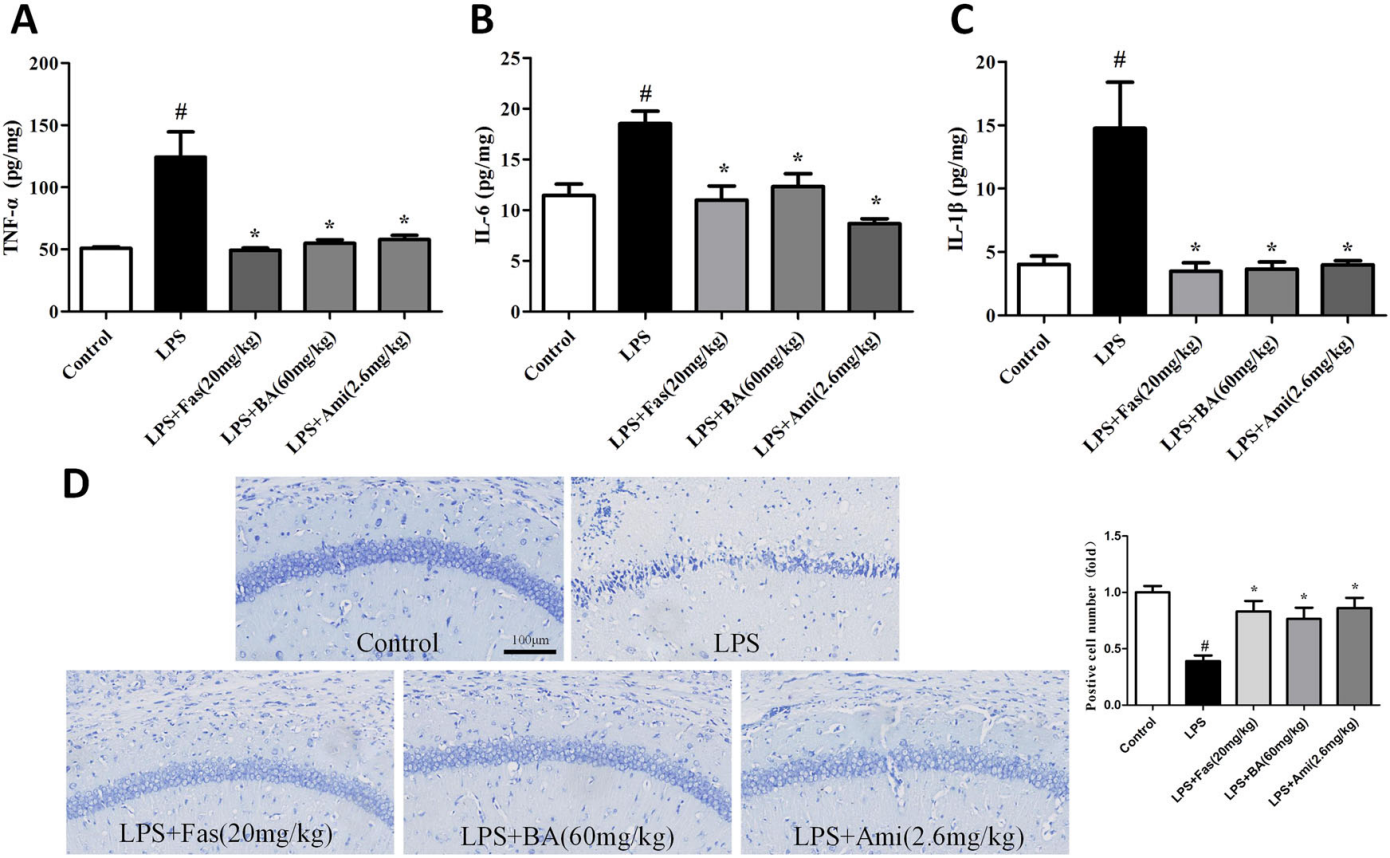
Effects of Na^+^/H^+^ exchanger 1 (NHE1) inhibition on lipopolysaccharide (LPS)-induced inflammation and apoptosis. **a**–**c** Effects of NHE1 inhibition by amiloride (2.6 mg/kg), baicalin (60 mg/kg), and fasudil (20 mg/kg) on pro-inflammatory cytokines including tumour necrosis factor-α, interleukin (IL)-6, and IL-1β in the hippocampus. **d** Representative photographs of Nissl-stained hippocampal CA1 region (magnification ×20, scale bar = 100 μm) and quantitative analysis of Nissl bodies in hippocampal CA1 region. Values are expressed as means ± SEM. ^#^*p* < 0.05 vs. control group; *p < 0.05 vs. LPS group

As shown in Fig. 6a–c, the levels of inflammatory cytokines tumour necrosis factor α (TNF-α), interleukin (IL)-6, and IL-1β in the hippocampus were remarkably elevated in the LPS group relative to the control group. However, pre-treatment with Ami (2.6 mg/kg), BA (60 mg/kg), and Fas (20 mg/kg) effectively downregulated the levels of TNF-α, IL-6, and IL-1β. In the CA1 region of the hippocampus, the number of Nissl-positive cells was significantly lower in the LPS group than in the control group. Administration of BA (60 mg/kg), Fas (20 mg/kg), and Ami (2.6 mg/kg) significantly increased the number of Nissl-positive cells (Fig. 6d).

### Effects of NHE1 inhibition on ROCK2/NHE1/calpain1 pathway in the hippocampus of LPS-treated mice

**Fig. 7:**
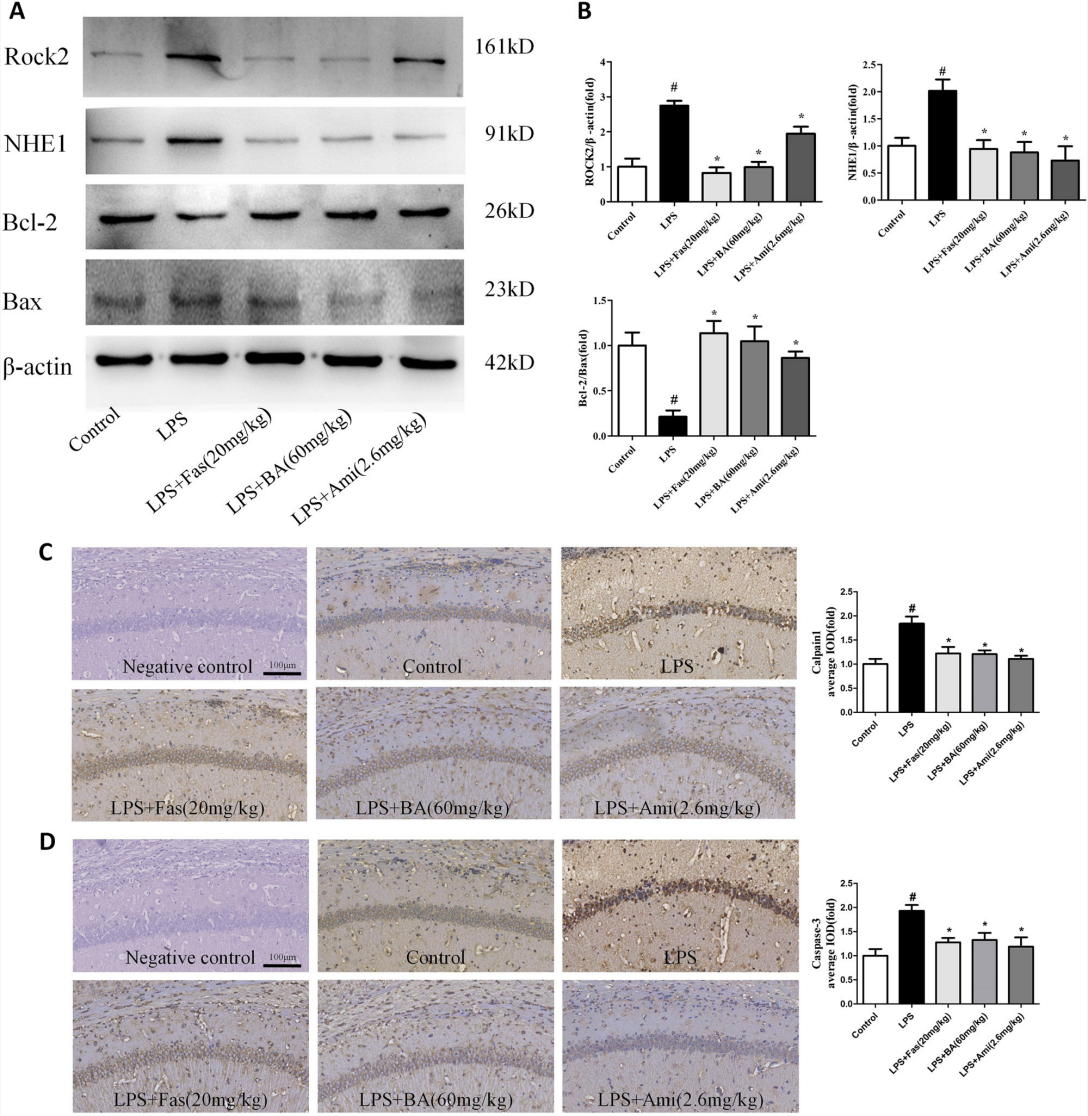
Effects of Na^+^/H^+^ exchanger 1 (NHE1) inhibition on ROCK2/NHE1/calpain1 pathway in the hippocampus of lipopolysaccharide (LPS)-treated mice. **a**, **b** The expression levels of Rho-associated kinase 2 and NHE1 and the Bcl-2-to-Bax ratio were detected through western immunoblotting. **c** Immunohistochemical staining of calpain1 in the CA1 region of the hippocampus. **d** Immunohistochemical staining of caspase-3 in the CA1 region of the hippocampus. Scale bar = 100 μm. Values are expressed as means ± SEM. ^#^*p* < 0.05 vs. control group; *p < 0.05 vs. LPS group

As described in Fig. 7a, b, the analytical results indicated that LPS significantly increased the levels of ROCK2 and NHE1 relative to those in the control group. Treatment with BA (60 mg/kg), Fas (20 mg/kg), and Ami (2.6 mg/kg) remarkably reversed these alterations. Furthermore, a decreased Bcl-2/Bax ratio was observed in the hippocampus of the LPS group, which was significantly reversed by BA (60 mg/kg,), Fas (20 mg/kg), and Ami (2.6 mg/kg) administration. Immunohistochemical results shown in Fig. 6c, d found significantly increased levels of calpain1 and caspase-3 in the hippocampal CA1 region of LPS mice relative to those in the control group. The administration of BA (60 mg/kg), Fas (20 mg/kg), and Ami (2.6 mg/kg) prominently suppressed the excessive activity of calpain1 and caspase-3.

### Effects of NHE1 inhibition on ROCK2/NHE1/calpain1 pathway in LPS-treated PC12 cells

**Fig. 8:**
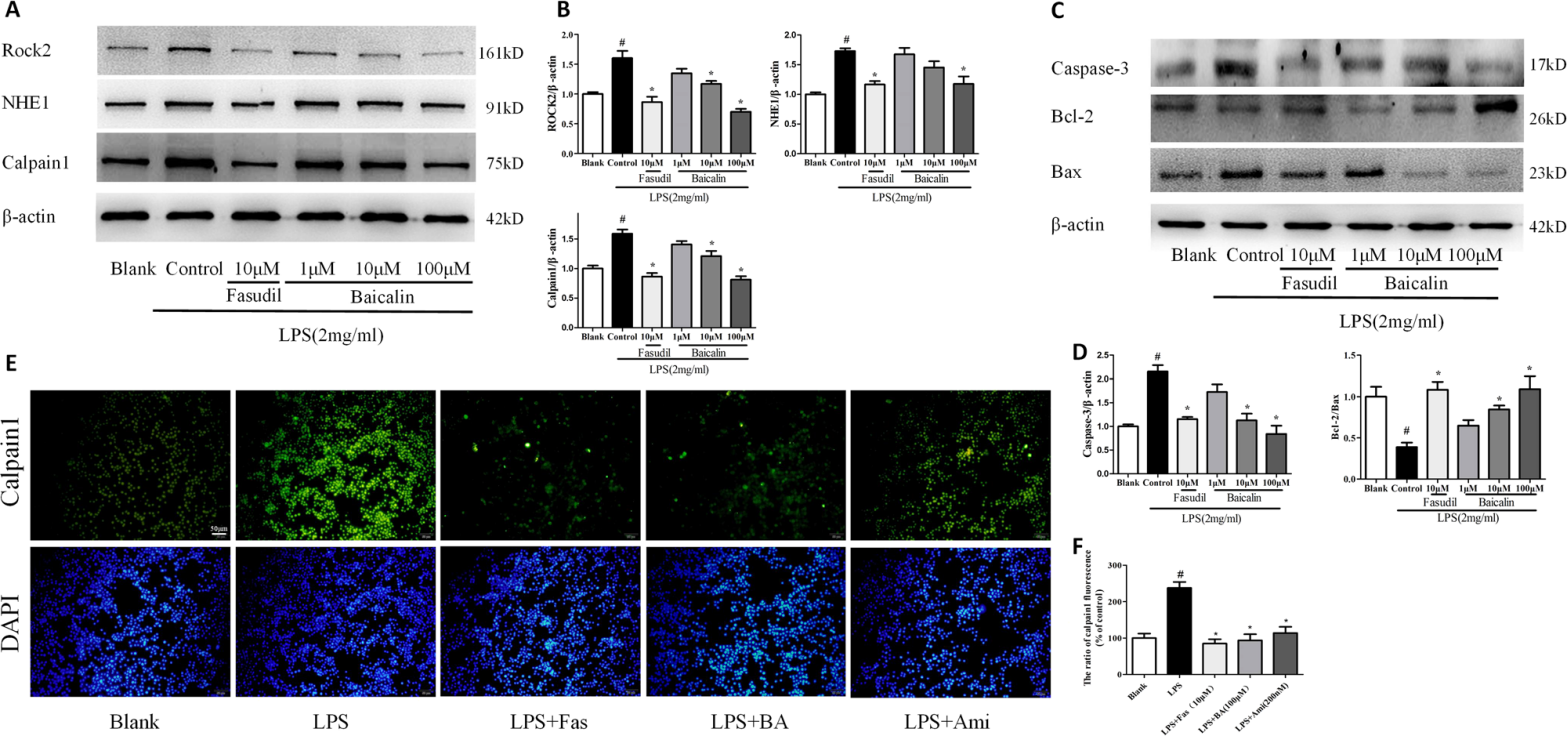
Effects of Na^+^/H^+^ exchanger 1 (NHE1) inhibition on ROCK2/NHE1/calpain1 pathway in lipopolysaccharide-induced PC12 cell. **a** Representative western blotting for Rho-associated kinase 2 (ROCK2), NHE1, and calpain1. **b** Effects of baicalin or fasudil treatment on the expression of ROCK2, NHE1, and calpain1 as seen on western blotting. **c** Representative western blot of caspase-3, Bcl-2, and Bax expression. **d** The expression levels of caspase-3, and the ratio of Bcl-2 to Bax. **e**, **f** The expression of calpain1 determined using immunofluorescence. Green represents calpain1 expression, and DAPI represents the nuclear stain. Scale bar = 50 μm. Values are expressed as means ± SEM. ^#^*p* < 0.05 vs. Blank group; *p < 0.05 vs. Control group

We further observed the effects of NHE1 inhibition in vitro. As shown in Fig. 8a, b, Fas (10 μM) and BA (100 μM) significantly suppressed the expression of ROCK2, NHE1, and calpain1. BA (10 μM) treatment inhibited the levels of ROCK2 and calpain. As shown in Fig. 8c, d, Fas (10 μM) and BA (10 μM, 100 μM) also significantly suppressed the expression of caspase-3 and increased the ratio of Bcl-2 to Bax. The effects of NHE1 inhibition through use of Ami, Fas, or BA on the expression of calpain were further evaluated using immunofluorescence. As shown in Fig. 8e, f, the expression of calpain1 was significantly increased after LPS challenge, which was reversed by Fas, BA, and Ami treatment.

### Effects of NHE1 inhibition on the CUMS-induced animal model of depression

**Fig. 9:**
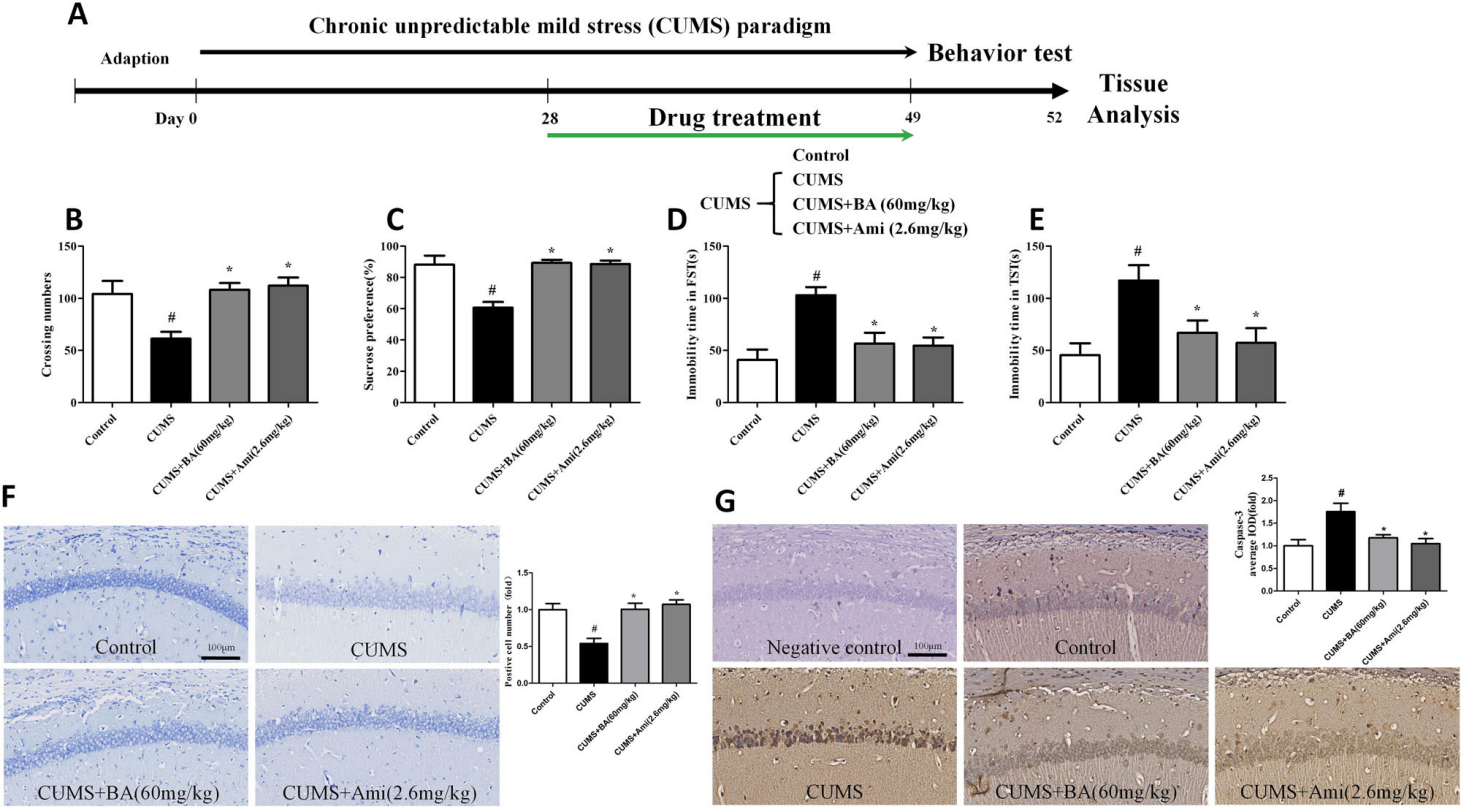
Chronic unpredictable mild stress (CUMS)-induced depressive-like behaviours in mice were reversed by chronic amiloride (Ami) treatment. **a** Schematic diagram of the experimental design for the CUMS paradigm. **b** Effects of Ami treatment on the number of crossing in CUMS-exposed mice in the open field test. **c** Effects of Ami treatment on the sucrose preference percentage in CUMS-exposed mice. **d** Effects of Ami treatment on the immobility time in CUMS-exposed mice in the forced swimming test. **e** Effects of Ami treatment on the immobility time in CUMS-exposed mice in the tail suspension test. **f** Representative photographs of Nissl-stained hippocampal CA1 region (magnification ×20, scale bar = 100 μm) and quantitative analysis of Nissl bodies in hippocampal CA1 region. **g** The immunohistochemical staining of caspase-3 in the CA1 region of the hippocampus. Scale bar = 100 μm. Values are expressed as means ± SEM. ^#^*p* < 0.05 vs. control group; *p < 0.05 vs. CUMS group

**Fig. 10:**
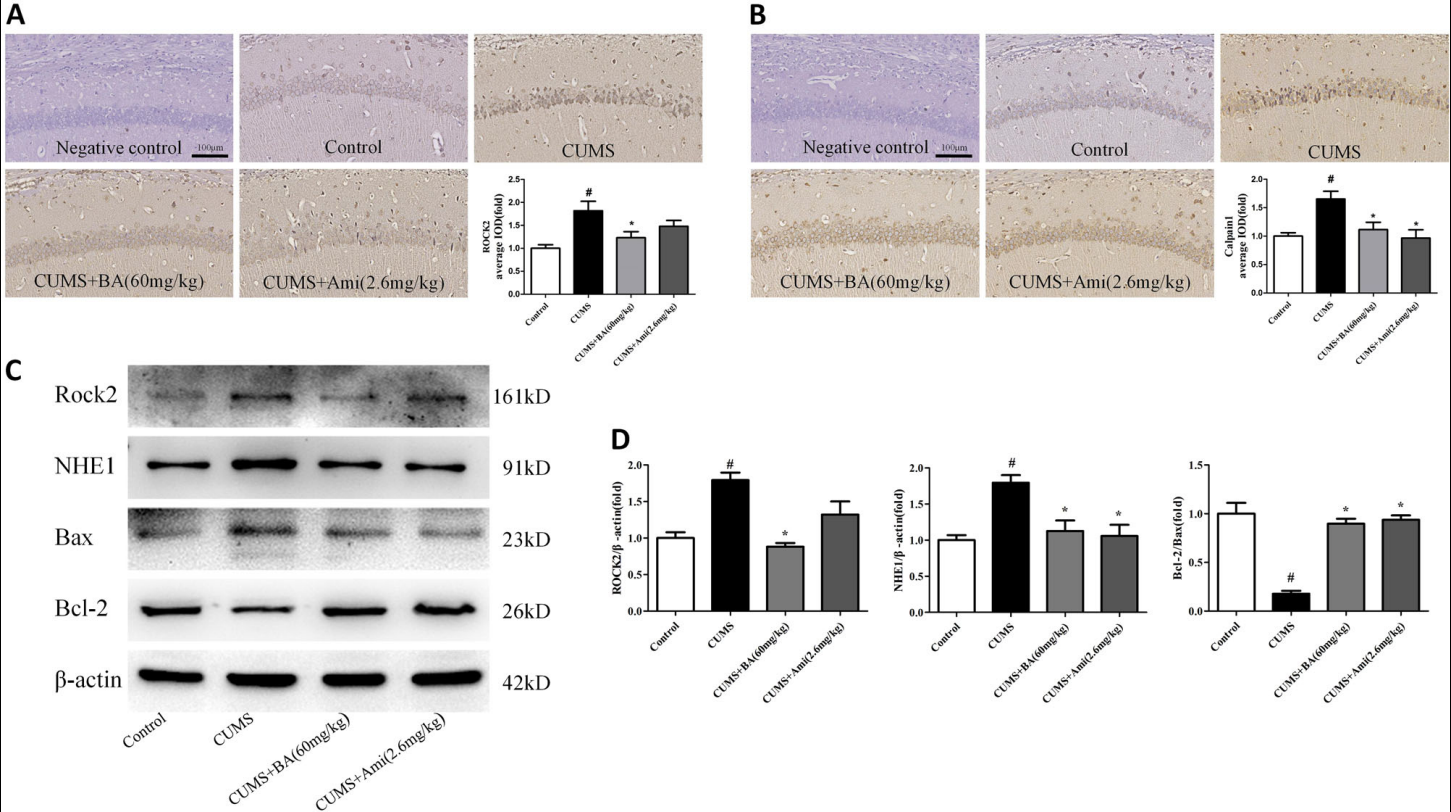
Amiloride (Ami) treatment attenuated the Na^+^/H^+^ exchanger 1 (NHE1)-regulated pathway in the hippocampus of chronic unpredictable mild stress (CUMS)-exposed mice. **a**, **b** Immunohistochemical staining of Rho-associated kinase 2 (ROCK2) and calpain1 in the hippocampal CA1 region of CUMS-exposed mice. **c** Representative western blot of ROCK2, NHE1, and Bcl-2 to Bax ratio. **d** The expression of ROCK2, NHE1, and Bcl-2 to Bax ratio. Scale bar = 100 μm. Values are expressed as means ± SEM. ^#^*p* < 0.05 vs. control group; *p < 0.05 vs. CUMS group

NHE1 inhibition by Ami was further evaluated in the CUMS-induced animal model of depression (Fig. 9a). As described in Fig. 9b–e, Ami (2.6 mg/kg) treatment significantly reversed the CUMS-induced decrease in locomotor activity and sucrose consumption and increase in immobility time in the FST and TST. Ami (2.6 mg/kg) treatment also restored the CUMS-induced decrease in the number of Nissl-positive cells in the hippocampal CA1 region (Fig. 9f) and increase in the caspase-3 (Fig. 9g), ROCK2, and calpain1 (Fig. 10a, b) in immunohistochemical experiments. Western blotting experiments show that Ami (2.6 mg/kg) treatment recovered the CUMS-induced increase in ROCK2 and NHE1 and decrease in the Bcl-2/Bax ratio (Fig. 10c, d).

## Discussion

Major depression is a highly debilitating and life-threatening mental disorder. However, due to their delayed efficacy, current antidepressant treatments cannot meet the demand of depressive patients. There is an unmet demand for new insights into the pathogenesis of depression, as well as the mechanisms of antidepressant therapeutics. In our present study, we first investigated the effects of ROCK2 inhibition in a CUMS-induced animal model of depression. Our results showed that ROCK2 inhibition via Fas or BA administration significantly alleviated CUMS-induced decreased sucrose consumption, reduced locomotor activity, and increased immobility time in the TST and FST. We further induced the activation of NHE1 in the LPS- and CUMS-induced animal models of depression and found that NHE1 inhibition via Ami administration inhibited the activation of the ROCK2/NHE1/calpain1 pathway and alleviated neural apoptosis.

We evaluated the effect of NHE1 in animal models of depression and explored ROCK2 inhibition as a neuroprotective strategy against neuronal apoptosis. The CUMS model is a well-established method to induce depressive-like symptoms in rodents through mimicking the stress environment of human depression^20^ and has been widely used to evaluate the efficiency of antidepressants. Mice in the CUMS group exhibited depressive-like behaviours that were consistent with previous studies^21^. As reported, stress exposure resulted in complex alterations, such as HPA axis dysfunction followed by neurodegeneration, causing symptoms of depression, such as anhedonia and increased immobility in the FST. However, our study observed that ROCK2 inhibition increased sucrose consumption and the number of crossings in the OFT and reduced immobility time in the TST and FST, indicating the beneficial effects of ROCK2 inhibition. LPS could selectively activate NHE1, which is related to activation of ROCK2 and excessive aggregation of Ca^2+^ in the cytoplasm. Moreover, LPS treatment triggered inflammatory responses, leading to neuronal apoptosis, which is also a well-established animal model of depression. Thus we employed LPS to induce depressive-like symptoms in mice to further demonstrate the pathogenesis of NHE1-regulated depression. Application of Ami, an NHE1 inhibitor, significantly reversed depressive-like behaviours induced by LPS challenge. Ami treatment also exhibited antidepressive effects in the CUMS-induced animal model of depression. The combination of these two animal models comprehensively investigated the beneficial effects of NHE1 inhibition in an animal model of depression and explained its upstream regulatory signalling.

Patients with depression undergo physical changes and alterations in behaviours in limbic brain regions, including the hypothalamus, hippocampus, and prefrontal cortex^22^. The hippocampus, in particular, is vulnerable to CUMS, leading to reduced volume, simplification of the dendritic arbour, and loss of dendritic spines^23^. Those patients with depression usually exhibit higher levels of pro-inflammatory cytokines in the hippocampus, which promote neuronal programmed death pathways including apoptosis, autophagy, and regulated necrosis^24^. In our research, Fas or BA treatment increased the number of Nissl bodies, indicating the integrity of CA1 region in the hippocampus, improved neuronal atrophy, and attenuated neuronal apoptosis in the hippocampus of CUMS- and LPS-induced animal models of depression. Furthermore, ROCK2 inhibition via Fas or BA administration suppressed the CUMS-induced increase in the expression of caspase-3 and reversed the decrease in Bcl-2/Bax ratio, which modulates mitochondrial membrane permeability and regulates mitochondrial apoptosis.

As reported in dopaminergic neurons, Bax cleaved by calpain led to the activation of caspase-3 and induced mitochondrial apoptosis. Thus we further investigated the role of calpain-related signalling in two animal models of depression. According to recent research, calpain inhibitors were shown to preserve mitochondrial function in cardiac tissue after ischaemia–reperfusion injury, an effect that was likely independent of any inhibition of caspases. The latest reports have exhibited the potential therapeutic effects of calpain inhibitors for neuronal protection in the treatment of neurodegenerative diseases like Alzheimer’s disease and cerebral ischemia. Thus calpain overexpression was proposed to play an important role in neuronal apoptosis in major depression. Furthermore, during neuronal death induced by salinomycin, calpain1 and calpain2 activated caspase-12, which in turn activated caspase-9 and its effector caspase-3 and resulted in neuronal apoptosis^25^. In our study, ROCK2 inhibition suppressed the expression of calpain in CUMS-treated mice, suggesting the involvement of calpain in depression. The causes of calpain overexpression are closely related to Ca^2+^ overaggregation, which is dependent on NHE1 expression. Our results show that CUMS induced the activation of NHE1 and ROCK2 inhibition attenuated NHE1-related signalling. Research on cerebral ischaemia has reported that the activation of NHE1 leads to caspase-3 activation, mitochondrial damage, and ischaemic apoptosis^26^. Therefore, calpain might act as a negative regulator of caspase processing and apoptosis. In endothelial cells, LPS stimulated NHE1 activation and increased Ca^2+^ concentration, which led to endothelial cell apoptosis^27^. LPS i.c.v., as a well-established animal model of depression, also activates NHE1, triggering inflammatory responses that lead to neuronal apoptosis^18^. By applying an inhibitor of NHE1, Ami, the depressive-like behaviours were significantly attenuated in the LPS-induced animal model of depression. These findings were consistent with results seen with atherosclerosis, where Ami administration inhibited NHE1 activity, thus attenuating LPS-accelerated damage in mice. The protective role of NHE1 inhibition was further illustrated in the CUMS-induced animal of depression to fully address the point. These observations suggest that NHE1 could modulate Ca^2+^ aggregation and might serve as a potential therapeutic target in depression.

Fas is one of the most thoroughly evaluated ROCK inhibitors and has been demonstrated to provide a beneficial effect in the management of neurological disorders, including depression, stroke, and Alzheimer’s disease^23,28^. BA is a natural ingredient with promising neuroprotective activity, including antidepressive activity, which has been reported to have potential in inhibiting ROCK activity^29^. Thus we adopted Fas, a ROCK inhibitor, and BA, a natural bioactive ingredient with inhibitory effects on ROCK2, to evaluate the antidepressant effects of ROCK2 inhibition in our present study. As shown from our results, CUMS exposure increased the levels of ROCK2 expression and NHE1 expression in the hippocampus of animals with depression-like behaviours. However, administration of BA and Fas decreased the levels of ROCK2 and NHE1 and improved neuronal apoptosis. These observations indicated that ROCK2 acted on NHE1 in the animal models of depression, which is consistent with previous reports that ROCK2 stoichiometrically phosphorylates NHE1 at threonine-653 in vitro^11^. These results illustrate the important role of ROCK2 with respect to NHE1 hyperactivity. Although ROCK2 is preferentially expressed in the brain, whereas ROCK1 is primarily expressed in the non-neuronal organs, a role for ROCK1 in depression cannot be completely ruled out.

LPS i.c.v. injection induced depressive-like behaviours and activation of NHE1. The application of Ami, an NHE1 inhibitor in the CUMS model, further indicated the clinical promise of NHE1 inhibition in the treatment of depression. Of note, LPS injection challenged the immune system, resulting in neuroinflammation, which is closely associated with neuronal apoptosis in the hippocampus. BA, Fas, and Ami pre-treatment suppressed the levels of inflammatory cytokines IL-1β, IL-6, and TNF-α, which improved neuroinflammatory responses in the hippocampus. These cannot eliminate the effect of microglia, which are the resident innate immune cells in the CNS and play an important role in neuroinflammatory response. In response to LPS stress, microglia transformed into a reactive inflammatory phenotype that could be cytotoxic and harmful to the neural microenvironment and might even trigger neurodegenerative and neuropsychiatric disease if excessive and prolonged neuroinflammation occured^30^. The NHE1 protein is also highly expressed in glioma-associated microglia; inhibition of microglial NHE1 activity downregulated microglia-derived factors, such as TNF-β and IL-6^31^. Thus the mechanisms of NHE1 inhibition on neurons and microglia still require further study.

**Fig. 11:**
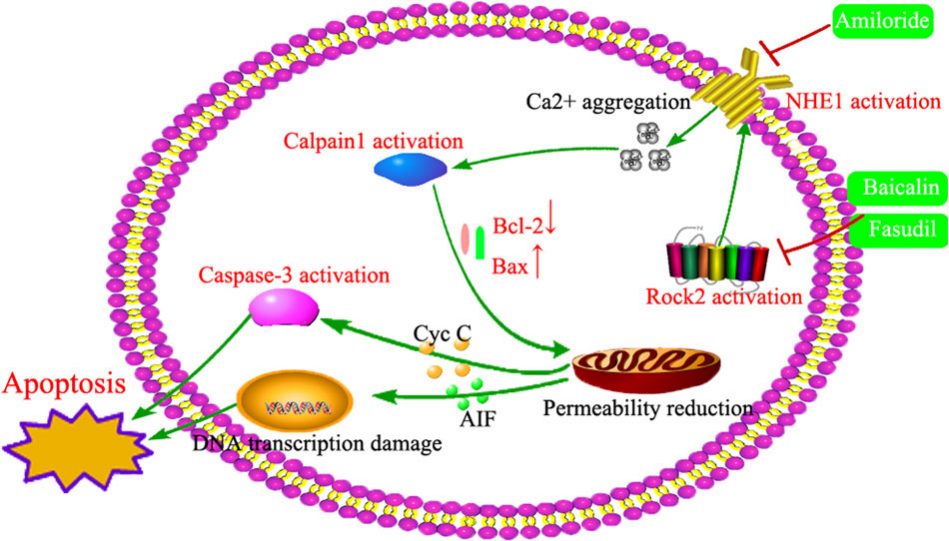
The proposed pathway for the role of the Na^+^/H^+^ exchanger 1 (NEH1) in depression. Chronic stress triggers NHE1 activation, induces calpain1-regulated apoptosis. NHE1 inhibition, by amiloride or through Rho-associated kinase 2 suppression by fasudil or baicalin, restored calpain1-regulated neural apoptosis and improved depressive-like behaviours in mice

Altogether, our results demonstrated that NHE1 inhibition relieved depressive-like behaviours in mice in both CUMS- and LPS-induced animal models of depression. Our study illustrated that NHE1 expression is of significance to the pathogenesis of depression and characterised its regulatory signalling through ROCK2 inhibition, which provided evidence that the ROCK2/NHE1/calpain1 signalling pathway could be considered a potential target in the treatment of depression. In conclusion, inhibition of ROCK2 may be a neuroprotective strategy against neuronal apoptosis, and NHE1 may be a potential therapeutic target in depression (Fig. 11).

## Materials and methods

### Animals

The male ICR mice used in our experiments were obtained from the Experimental Animal Center in Jiangsu Province (Nanjing, China). The mice weighing 20–22 g were housed in the animal centre at a consistent ambient temperature (23 ± 2 °C) with a 12-h light/dark cycle and free access to food and water. Mice had 3 days without any experiments for environmental adaptation. All animal experiments were strictly performed in accordance to the Provision and General Recommendation of Chinese Experimental Animals Administration Legislation and approved by the Science and Technology Department of Jiangsu Province.

### Drugs and reagents

BA was obtained from Xi’an Kailai Co, Ltd.; Fas was purchased from Apex Bio Technology LLC; Ami and LPS were purchased from Sigma-Aldrich Co., Ltd. (St. Louis, USA). The bicinchoninic acid assay (BCA) kit was procured from Beyotime Institute of Biotechnology Co., Ltd. IL-1β, TNF-α, and IL-6 enzyme-linked immunosorbent assay (ELISA) kits were supplied by Nasheng Biological Technology Co., Ltd. Rabbit-ROCK2 antibody (20248-1-ap), rabbit-calpain1 antibody (10538-1-ap), and rabbit-caspase-3 antibody (19677-1-ap) were supplied by Proteintech Group, Inc. Rabbit-NHE1 antibody (DF9933) was supplied by Affinity. Rabbit-Bcl-2 (Ab32124) antibody was supplied by Abcam plc. Mouse-Bax antibody (Sc-20067) was supplied by Santa Cruz Biotechnology, Inc. Mouse-anti-β-actin (BS6007M) was supplied by Bioworld Technology, Inc.

### CUMS model

The CUMS procedure was conducted according to the method reported previously^8^. All stressors were randomly scheduled and changed every day to maintain an unpredictable procedure. All stressors are shown in Fig. 1a, and the experimental procedure for CUMS is exhibited in Fig. 2a. The mice were treated with drugs for the next 21 days after being exposed to a 28-day protocol of CUMS, during which the CUMS procedure was performed every day. The animal model of depression was successfully established after a 28-day CUMS protocol, as indicated by the significant decrease of sucrose consumption in all the CUMS groups. (Data are shown as Fig. S1 in supplemental materials.). In this procedure, male ICR mice were randomly divided into six groups after environmental adaptation: control group (saline, intragastric gavage (ig)), control+BA (60 mg/kg/day, ig) group, CUMS group (saline, ig), CUMS+Fas (20 mg/kg/day, ig) group, CUMS+BA (30 mg/kg/day, ig) group, CUMS+BA (60 mg/kg/day, ig) group (Fig. 1b). In the experiment of Ami treatment on CUMS-induced depressive-like behaviours in mice, the groups were as following: control group (saline, ig), CUMS group (saline, ig), CUMS+BA (60 mg/kg/day, ig) group, CUMS+Ami (2.6 mg/kg/day, ig) group (Fig. 9a).

### LPS exposure model and inflammatory cytokine measurements in the hippocampus

The experimental procedure for the LPS model, which has been previously reported, is shown in Fig. 5a. One microlitre saline or 1 μl of 10 mg/ml LPS in saline was i.c.v. injected into the mice at a constant rate of 0.3 μl/min at the following coordinates: −2.5 mm dorsal/ventral, −1.0 mm lateral, and −0.5 mm anterior/posterior from the bregma^18^. After injection, the needle stayed in the brain for 5 min in case of any leakage of LPS. Ami, a antagonist of NHE1, and Fas were used to examine the ROCK2/NHE1 signalling pathway. In this procedure, male ICR mice were randomly divided into five groups after adaptation: control group, LPS group, LPS+Fas (20 mg/kg/day, ig) group, LPS+BA (60 mg/kg/day, ig) group, LPS+Ami (2.6 mg/kg/day, ig) group (Fig. 5a). The concentrations of IL-1β, IL-6, and TNF-α in the hippocampus were tested using ELISA kits according to the manufacturer’s instructions.

### Sucrose preference test

At first, every cage was given two bottles for 12 h adaptation, one contained 1% sucrose solution, and another contained tap water. Each mouse was then housed in a single cage with water and were food deprived for 12 h. After deprivation, each mouse was given two new bottles, again one with 1% sucrose water and one with tap water. All mice had equal access to the two bottles. During the test, we would change the positions of the two bottles in case of possible side-preference (SP) effects. SP was calculated as SP (%) = sucrose water intake (g)/(sucrose water intake (g) + tap water intake (g)) × 100%.

### Open field test

The experimental apparatus was a metallic circular enclosure with 12 equal arenas. The mice were individually placed in the centre of the enclosure and allowed to explore freely for 6 min in a quiet environment. The number of squares crossed by the mice were recorded during the last 4 min.

### FST and TST

For the FST, the mice was forced to swim in 25 ± 2 °C water for 10 min 1 day before the test. The test apparatus comprised a glass cylinder 20 cm in height and 14 cm in diameter. The mice were forced to swim in the apparatus for 6 min, and the duration of immobility was recorded for the last 4 min. When the mice were floating with some small movements necessary to remain afloat and without struggling, they were considered to be immobile. In the TST, each mouse was suspended, approximately 1 cm from the tail tip, from the test instrument using adhesive tape for 6 min. The total immobility time was recorded for the last 4 min. When the mice was hanging passively without struggling, it was considered immobile.

### Immunohistochemistry

Mice were euthanised with 4% chloral hydrate intraperitoneally and the brain tissues were then quickly removed on ice and immersed in the fixative of 10% formalin. The brains were then sectioned, stained, and incubated in the corresponding primary antibodies for 12 h at 4 °C. After washing with phosphate-buffered saline (PBS), the sections were incubated with a biotinylated goat anti-rabbit antibody for 1 h at room temperature. In order to exclude false-positive results, we established negative control groups that used PBS instead of primary antibody. The images of the hippocampus were captured using the Digital Image Scanner (Hamamatsu, Zanoomer 2.0RS). The location of the CA1 region to be evaluated was indicated by a rectangle with a black border, as shown in Fig. 3a. The protein expression levels of the indicated hippocampal CA1 region (magnification ×20) were determined for each animal. The quantitative analysis was carried out using the Image Pro-Plus version 6.0 (IPP6, Media Cybernetics Inc., MD, USA) software to determine the positive signal using average integrated optical density (IOD) of the hippocampal CA1 section within the chosen area of interest (AOI). The values of average IOD (IOD SUM/Area SUM) were used for statistical analysis.

### Nissl staining

The procedure was the same as that detailed in the “Immunohistochemistry” section. Toluidine blue was used to stain sections for neuronal cell bodies. The sections were then rinsed in distilled water and differentiated in 95% ethanol until the desired colour was attained. After staining using 0.5% eosin, the sections were mounted, air-dried, dehydrated, and coverslipped. The images of the CA1 region in the hippocampus were also captured by the Digital Image Scanner (Hamamatsu, Zanoomer 2.0RS). The survived hippocampal neurons of the CA1 region (magnification ×20) were counted for each animal by investigators blinded to the study groups.

### Cell culture

PC12 cells were cultured in Dulbecco’s modified Eagle’s medium basic medium containing 10% foetal bovine serum, 100 U/ml penicillin, and 100 μg/ml streptomycin, in a humidified atmosphere containing 5% CO_2_ at 37 °C. PC12 cell damage was induced using LPS at a concentration of 2 mg/ml as previously described. In the present investigation, PC12 cells were exposed to LPS (2 mg/ml) for 10 h to induce inflammatory stress injury in this study. BA (1 μM, 10 μM, and 100 μM), Fas (10 μM), or Ami (200 nM) was added 2 h prior to LPS stimulation. All experiments were carried out after cells were seeded at an appropriate density.

### Immunofluorescence

The supernatant was discarded from the six-well plates after drug treatments and LPS stimulation. Rabbit anti-calpain1 at 1:200 dilution was added, and the cells were incubated overnight at 4 °C. Tris-buffered saline containing Tween 20 was used to wash three times, and an antibody dilution solution containing Cy3-labelled goat anti-rabbit IgG was added. The plates were covered with a black box for 2 h at room temperature. Cells were then incubated with DAPI (1:50) at 37 °C for 5 min after PBS wash. Finally, cells were mounted in mounting medium and visualised under a fluorescence microscope.

### Immunoblotting

The protein concentration was determined using a BCA protein assay kit. Equal protein amounts were separated on sodium dodecyl sulfate-polyacrylamide gel electrophoresis gels and transferred to the polyvinylidene difluoride membranes. The membranes were blocked with 5% skimmed milk and then incubated with primary antibody for 12 h at 4 °C. After washing in PBS-Tween, the membranes were incubated with secondary antibodies. Immunoreactivity was detected using an enhanced chemiluminescence detection system and visualised with an imaging system (Bio-Rad, Hercules, CA, USA). β-Actin was used as the control, and ROCK2, NHE1, calpain1, caspase-3, Bcl-2, and Bax were detected. The images were quantified using the Image-J software.

### Statistics

Data are expressed as the mean ± S.E.M. Significant differences were analysed using one-way analysis of variance followed by Tukey’s test. The value *p* < 0.05 was considered statistically significant.

## Supplementary information

Figure S1

Supplementary figure legends
