# Supplementary figures and images for: Suppressing the Na+/H+ exchanger 1: a new sight to treat depression
Source: Cell Death Dis. 2019 May 8;10(5):370. doi: 10.1038/s41419-019-1602-5 (PMC6506522; doi:10.1038/s41419-019-1602-5)

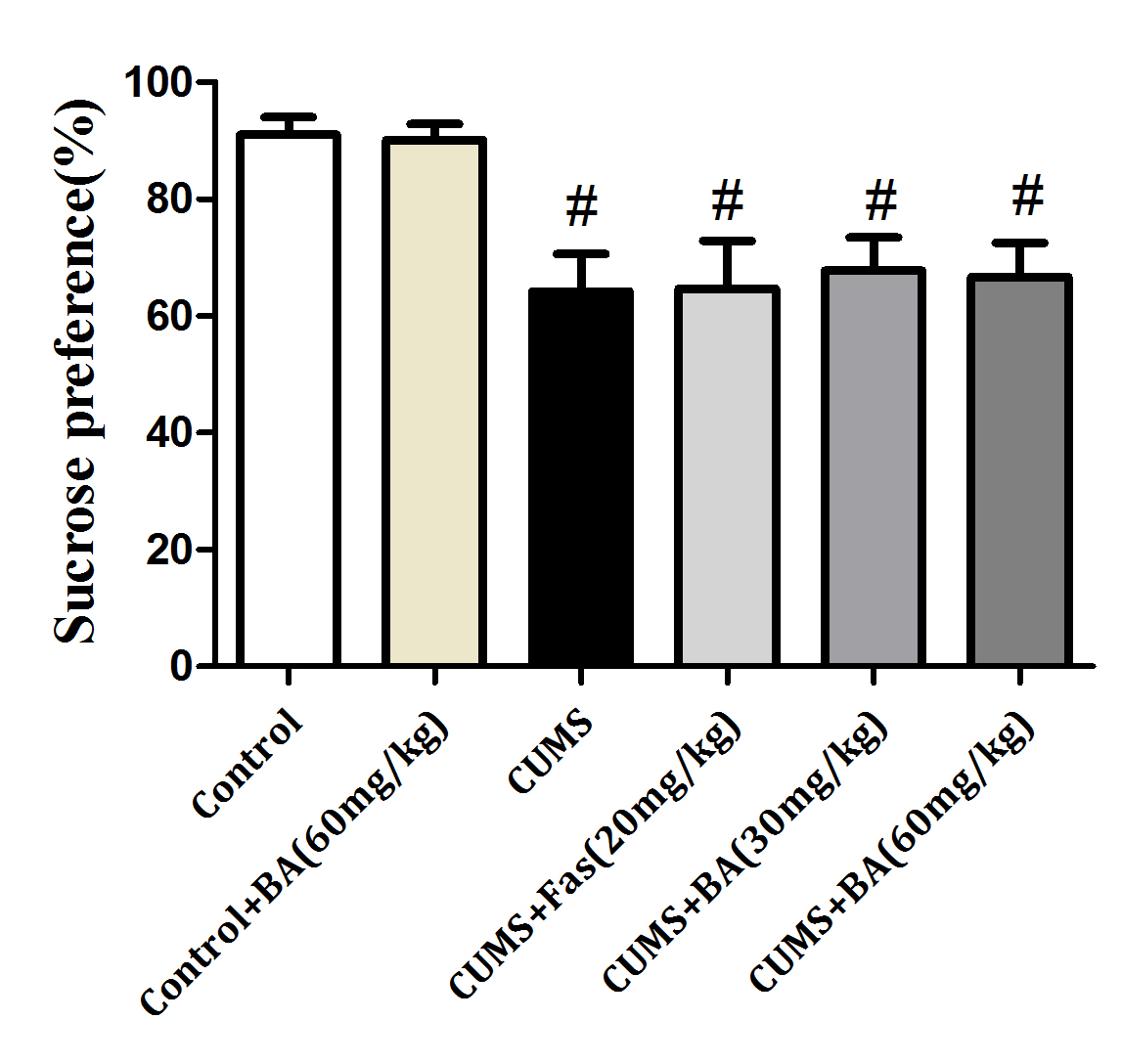

Supplement: Supplementary file 1 — Figure S1 [file 41419_2019_1602_MOESM1_ESM.jpg]
