# Supplementary material for: Suppressing the Na+/H+ exchanger 1: a new sight to treat depression
Source: Cell Death Dis. 2019 May 8;10(5):370. doi: 10.1038/s41419-019-1602-5 (PMC6506522; doi:10.1038/s41419-019-1602-5)
Supplement: Supplementary file 2 — Supplementary figure legends [file 41419_2019_1602_MOESM2_ESM.docx]

Supplementary figure legends

Fig. S1 The sucrose consumption was detected after a 28-day CUMS protocol.
